# How citizen science could improve species distribution models and their independent assessment

**DOI:** 10.1002/ece3.7210

**Published:** 2021-03-10

**Authors:** Florence Matutini, Jacques Baudry, Guillaume Pain, Morgane Sineau, Joséphine Pithon

**Affiliations:** ^1^ BAGAP INRAE, Institut Agro ESA Angers France; ^2^ BAGAP INRAE, Institut Agro ESA Rennes France; ^3^ URCPIE Pays de la Loire Nantes France

**Keywords:** amphibians, biodiversity conservation, data culling, data filtering, external evaluation, habitat suitability modeling, sampling effort

## Abstract

1. Species distribution models (SDM) have been increasingly developed in recent years, but their validity is questioned. Their assessment can be improved by the use of independent data, but this can be difficult to obtain and prohibitive to collect. Standardized data from citizen science may be used to establish external evaluation datasets and to improve SDM validation and applicability.
2. We used opportunistic presence‐only data along with presence–absence data from a standardized citizen science program to establish and assess habitat suitability maps for 9 species of amphibian in western France. We assessed Generalized Additive and Random Forest Models’ performance by (1) cross‐validation using 30% of the opportunistic dataset used to calibrate the model or (2) external validation using different independent datasets derived from citizen science monitoring. We tested the effects of applying different combinations of filters to the citizen data and of complementing it with additional standardized fieldwork.
3. Cross‐validation with an internal evaluation dataset resulted in higher AUC (Area Under the receiver operating Curve) than external evaluation causing overestimation of model accuracy and did not select the same models; models integrating sampling effort performed better with external validation. AUC, specificity, and sensitivity of models calculated with different filtered external datasets differed for some species. However, for most species, complementary fieldwork was not necessary to obtain coherent results, as long as the citizen science data were strongly filtered.
4. Since external validation methods using independent data are considered more robust, filtering data from citizen sciences may make a valuable contribution to the assessment of SDM. Limited complementary fieldwork with volunteer's participation to complete ecological gradients may also possibly enhance citizen involvement and lead to better use of SDM in decision processes for nature conservation.

## Graphical abstract

To develop new scientific and participatory approaches might better integrate biodiversity issues into decision‐making processes. In this study, we used different datasets to establish and assess habitat suitability maps for 9 species of amphibian in France. We compared different SDM as well as the effect of integrating parameters relating to sampling effort. We assessed the models’ performance by (1) internal cross‐validation or (2) external validation using an independent dataset from citizen science monitoring strategically completed. The AUC values obtained with cross‐validation were much higher for all species than with external evaluation and models integrating sampling effort performed better with external validation methods. Data from citizen science may help to get valuable external dataset to the assessment of SDM.

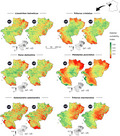

## 1 INTRODUCTION

In the current context of biodiversity loss, a stronger relationship between conservation science and citizen participation could help to make conservation actions more effective (Forrester et al., 2017; Lewandowski & Oberhauser, 2017). Availability of data from citizen sciences has considerably increased over the past few decades (Dickinson et al., 2010; McKinley et al., 2017). This data have great potential because (a) large quantities of data can be collected over large areas, which would be difficult and expensive for researchers to collect; (b) data may be collected over long time periods, which is especially useful for studying the effects of climate and landscape changes on population dynamics at large scales; (c) citizens are involved in the research process, thereby gaining knowledge, and their involvement might lead to improved implementation of biodiversity conservation action (Dickinson et al., 2010; McKinley et al., 2017). However, quality of data from participatory sciences is heterogeneous and different methods have been developed to boost data accuracy and account for bias, including interactive project development, volunteer training, expert data validation, and statistical modeling improvement (Kosmala et al., 2016). Although researchers have been skeptical about the value of datasets from citizen science, recent publications show that some could be as valid as data collected by professional scientists (Kosmala et al., 2016). This is conditional on such data being judged in context (i.e. according to the sampling methods used, program objectives, and applications) on the use of rigorous data sorting and analyses (Isaac et al., 2014; Robinson et al., 2020; Steen et al., 2019).

Opportunistic presence‐only data collected by citizens at large scales have contributed to the expansion of species distribution models (SDM) over the past twenty years, particularly for biological conservation applications (Guisan & Thuiller, 2005). The validity of presence‐only SDM is, however, increasingly questioned as well as their applicability (Barve et al., 2011). Presence‐only data come from different source databases reduced to simple species presence records and mostly collected in an unstandardized way by volunteers. In contrast to presence–absence data, they are abundant but have poor quality, few metadata and come from different sources (Robinson et al., 2020). This introduces numerous sources of bias that need to be assessed and accounted for in modeling processes (Guillera‐Arroita et al., 2015; Phillips et al., 2009). Common problems are heterogeneous sampling effort, conditions and methods, imprecise spatial and temporal resolutions, and different levels of expertise among observers (Dickinson et al., 2010; McKinley et al., 2017; Phillips et al., 2009; Schulman et al., 2007). Different methods have been developed to correct these biases, including sorting or weighting presence‐data to reduce identification errors and pseudo‐replication linked to sampling effort (Guisan & Theurillat, 2000; Phillips et al., 2009) and/or using sampling effort assumptions to establish pseudo‐absence sampling strategies (Barbet‐Massin et al., 2012). Understanding the structure and intensity of sampling effort in space is essential to determine whether an undetected species is truly absent. For example, it may be conditioned by site accessibility (Kadmon et al., 2004; Phillips et al., 2009), site attractiveness, or observer distribution (Phillips et al., 2009; Robinson et al., 2018). Not accounting for heterogeneous sampling effort or using erroneous assumptions to define it can lead to overassessment of model accuracy and/or false interpretation (Guillera‐Arroita et al., 2015; Phillips et al., 2009; Schulman et al., 2007).

SDM validation is challenging (Vaughan & Ormerod, 2005) but is a crucial step for applying results to conservation objectives. There is still debate about SDM validity, especially when presence‐only data are used to calibrate models (Barve et al., 2011). Using data with the same spatial bias to calibrate and assess a model tends to overestimate prediction accuracy, by modeling observation processes more than ecological processes therefore producing erroneous results. Currently, testing model accuracy with a fully independent dataset is considered the most robust method for assessing SDM (Araujo et al., 2005; Guisan et al., 2017). However, obtaining an external dataset for large‐scale studies is often cost prohibitive and exploiting data from standardized citizen science programs may in some cases provide the solution. For example, Robinson et al. (2020) have shown that using filtered large‐scale citizen science data for SDM calibration can improve model accuracy. Alternatively, detection–nondetection data from more standardized citizen sciences programs which are rarer than opportunistic data but have higher quality could provide presence–absence sets for external validation of presence‐only SDM. In addition, using presence‐only and presence–absence data at different stages of the modeling process could be a method for combining different datasets with heterogeneous quality which is a current challenge to improve SDM validity (Robinson et al., 2020; Zipkin & Saunders, 2017).

Amphibians are among the most threatened taxa in the world with rapid and widespread population declines (Stuart et al., 2004). They are particularly sensitive to fragmentation and habitat loss (Cushman, 2006) because they need different resources during their life cycle involving movements (seasonal migration and dispersion) between aquatic sites (usually ponds) and terrestrial areas (Cushman, 2006; Sinsch, 1990). Many citizen science programs have been initiated for monitoring amphibian species (De Solla et al., 2005; Schmeller et al., 2009), and data collected have been used in some conservation studies to describe population trends (Petrovan & Schmidt, 2016), road effects (Cosentino et al., 2014), climate change (Préau et al., 2019), and large‐scale species distributions (Brown et al., 2016). Despite abundant literature on amphibian ecology and the availability of several citizen science databases, few studies have attempted predictive amphibian distribution models at large scales (Brown et al., 2016; Graham & Hijmans, 2006; Préau et al., 2019). Therefore, amphibian data could be suitable for testing the capacity of different types of citizen data (presence–absence or opportunist) for calibrating and assessing SDM.

Here, we compare the predictive performance of presence‐only SDM for nine amphibian species using different types of data (*internal* presence‐only or *external* presence–absence) from citizen science programs for their assessment. We also test the opportunity to use filtered standardized citizen science data to constitute the independent dataset for external evaluation. We hypothesized that (1) the type of data used for validation (internal or external) would influence the assessment of model accuracy; (2) standardized citizen science datasets might be used as independent data for external evaluation of SDM using data filters and/or complementary fieldwork.

## 2 MATERIALS AND METHODS

### 2.1 Study area

Our study was performed in Pays de la Loire (western France), a region covering 32,082 km^2^ with low relief and bordering on the Atlantic Ocean to the west. The region has an extensive hydrographic network organized around the River Loire and its tributaries, influencing local climate and landscape configuration. Agricultural landscapes dominate the region and traditional hedgerow network landscapes associated with extensive livestock farming are recognized for their conservation value. Such mosaics of small pastures delimited by hedgerows and small woods and generally associated with dense pond systems (Baudry et al., 2000) are favorable for many organisms including endangered species such as some amphibians species (Boissinot et al., 2019). With 21 known species (for 43 species recorded in France), the region has a high responsibility for the preservation of amphibians and their habitats, including traditional hedgerow landscape and wetlands.

### 2.2 Biological data

We studied habitat suitability of 9 amphibian species: *Bufo spinosus, Hyla arborea, Rana dalmatina, Rana temporaria, Triturus cristatus, Triturus marmoratus, Lissotriton helveticus, Salamandra Salamandra,* and *Pelodytes punctatus*. Two types of amphibian data were used: (1) opportunistic data from a citizen database with presence‐only records for model calibration and internal evaluation and (2) standardized detection–nondetection data from a citizen science program and complementary field work for external evaluations. A more detailed description of the datasets and complementation strategies is available in Appendix S1.

#### 2.2.1 Opportunistic presence data (calibration and cross‐validation dataset)

We accessed presence‐only occurrences from a regional database for the period 2013–2019. 86% of the dataset was collected by citizens and recorded online (website or associated mobile application) and 14% by various professional organizations involved in nature protection. All data were compiled for the regional Atlas of amphibians by a French nongovernmental organization (French BirdLife partner—LPO). See Appendix S1: Table S1 for data sources. We retained only species with enough data according to number of predictors used (i.e. at least 477 presence cells; see Appendix S1: Table S2). We selected only precise GPS records (precision of the observation under 50 m), and we checked all data for anomalies in geographical location or species identification.

For each species, we sorted data to reduce spatial autocorrelation by projecting presence data on a 500 m resolution grid and retaining only cells containing at least one occurrence as presence cells for the analyses (Guisan & Theurillat, 2000). We chose a 500 m resolution as it is the mean size of species’ home ranges (Semlitsch & Bodie, 2003). Finally, we excluded all opportunist data intersecting cells used for external validation described below to increase independency between calibration and validation sets.

#### 2.2.2 Standardized detection–nondetection data (external validation datasets)

**FIGURE 1:**
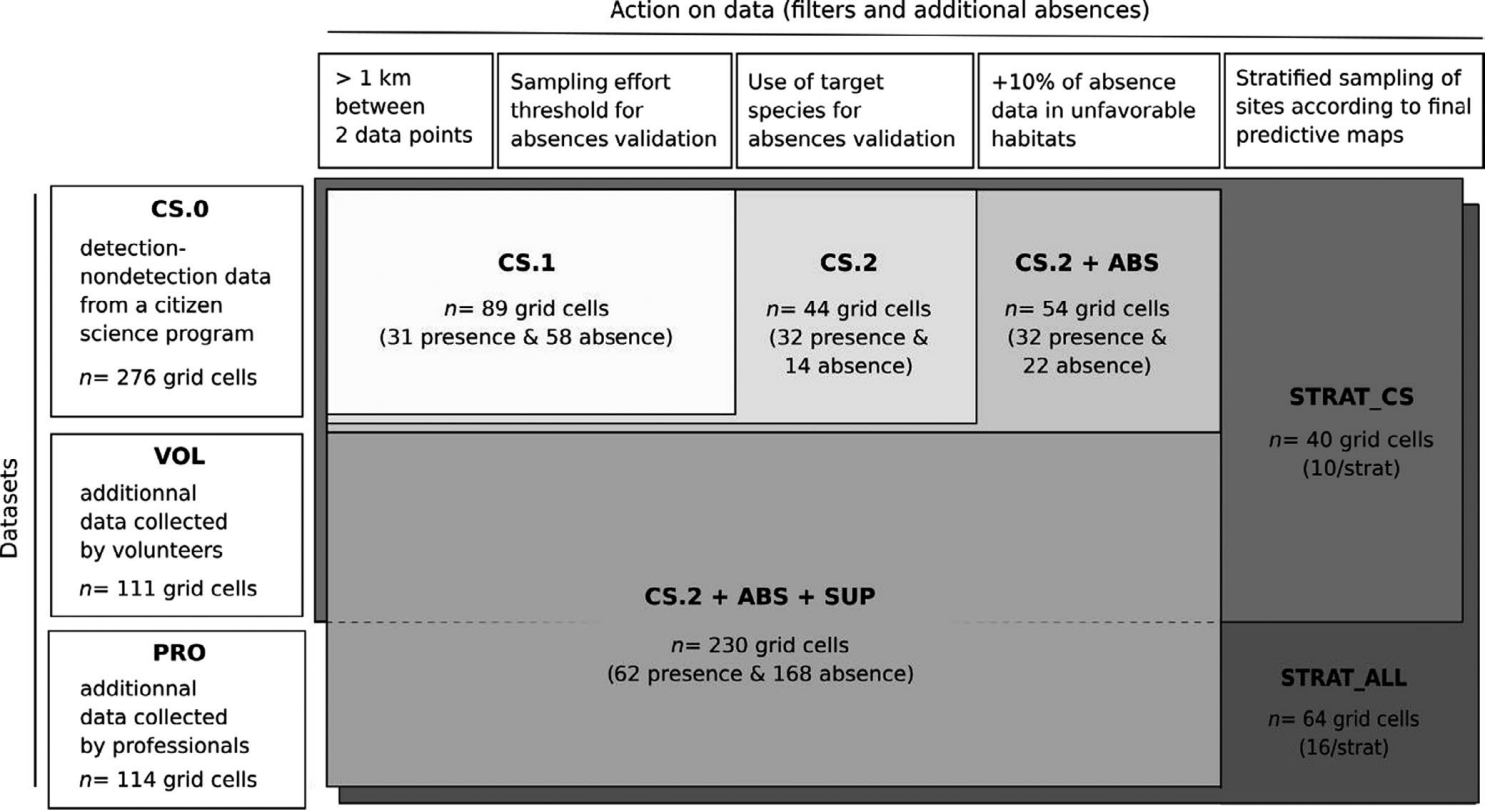
Description of datasets, filters and complementation used for external evaluation derived. Number of data (*n* grid cells) given for *T. marmoratus*, for 1 iteration only and s3. CS: data derived from the citizen science data set; SUP: additional data collected by volunteers (VOL) and professionals (PRO)

For external validation, we first extracted *detection–nondetection* amphibian data for the period 2013–2019 collected as part of a citizen science program called “Un Dragon dans mon Jardin” (Appendix S1: Section 1.2). We retained 576 sites which were monitored at least 3 times between February and June during at least one year and following a standard method commonly used for amphibian community surveys (Boissinot et al., 2019). We called this dataset *CS.0* (see Figure 1). Some large areas of the region were not sampled due to lack of observers so that data were clustered near cities, with spatial autocorrelation. Therefore, with help from volunteers, we completed this dataset with some additional fieldwork and applied filters.

To complete and filter *CS.0*, different strategies were used. First, we organized complementary fieldwork in 2018 and 2019 to complete two landscape gradients (woody element density and pond density) which are two variables known to strongly affect amphibian distribution and which are relevant in our regional context. All sites were selected randomly but so as to maximize and decorrelate the two landscape gradients in different areas (see Appendix S1: Section 1.3). In total, 263 sites were monitored: 132 sites by experts in 2018–2019 (called *PRO*, see Figure 1) and 131 by 75 volunteers in 2019 (called *VOL*, see Figure 1). All data (*CS.0*, *PRO* and *VOL*) were projected on the same 500 m resolution grid. One further problem, common in citizen science programs (Geldmann et al., 2016), is that only aquatic sites are surveyed while areas known to be very unsuitable for amphibians such as urban areas and intensive agriculture are generally excluded. To reduce this source of bias, we randomly selected 5% more 500 m grid cells in totally urbanized areas without aquatic sites and 5% more grid cells in homogeneous croplands without trees or ponds and we attributed “absence” values to each after field checks (called *ABS*, see Figure 1). These landscapes represent 9% of the total area of the region.

Second, we applied different filter combinations to establish subsets from *CS.0*, *PRO,* and *VOL* (see Figure 1):

1. A minimum distance of 1 km between two grids containing data;
2. Threshold values to validate nondetection as absence data and exclude under‐sampled sites, defined as a minimum sampling effort required to detect a species based on the species’ detectability group and observer level of expertise. Four species detectability groups were defined from occupancy studies in France (Boissinot, 2009; Petitot et al., 2014) and the UK (Sewell et al., 2010). Observers were classed as either *expert*, *intermediate,* or *novice* using 3 criteria: number of years of participation, number of species observed and permit holder for amphibian capture. A “*novice*” observer was considered more likely to miss or misidentify a species which was actually present than an “*expert*” observer for the same considered survey effort. In addition, novice observers did not use sampling nets, influencing detectability, in particular of Urodeles. Based on our observer classes and sampling methods used (e.g., acoustic, visual and/or fishing), we set threshold values for the minimal sampling effort needed to validate absence data, depending on species detectability (see Appendix S1: Section 1.4 for details) and according to the results from Boissinot (2009) for minimum sampling effort needed to detect a focal species (with 95% probability) in a similar biological and landscape context.
3. Target species to valid nondetection as absence, as recommended by Phillips et al. (2009). So, if *species A* with the same detectability as *species B* is detected at a site, then *species B* is likely to be truly absent (see Appendix S1: Section 1.5 for target species list).
4. Stratified sampling on final prediction maps (see STRAT_CS and STRAT_ALL in Figure 1). We established independent datasets, stratified by model predictions for each species (Newbold et al., 2010; Guisan et al., 2017) with equal random sampling of validation cells with presence or absence data in 4 habitat suitability classes predicted (i.e. [0;0.3[, [0.3;0.5[, [0.5;0.7[ and [0.7;1]). We obtained an equal number of validation cells by predicted suitability class (see 1.4.3 for predictive map used).

### 2.3 Environmental dataset and variables

We assembled environmental data relevant to amphibian ecology and of importance in the study region (Guisan et al., 2017). A more detailed description of variables with associate references is available in Appendix S2: Table S1.

Bioclimatic variables were accessed from a compilation of climate data for the period 1950–2000 at a spatial resolution of 5 km^2^ (Hijmans et al., 2005). An altitude variable was derived from the U.S. Geological Survey's Hydro‐K dataset, at the same spatial resolution. We performed a principal‐components analysis (PCA) on 11 bioclimatic variables relevant for amphibians and the altitudinal layer to produce 2 uncorrelated axes (see Appendix S2: Table S4 and Figure S2). Land‐cover data were downloaded from the highly detailed vector database OCS GE 1.1 (IGN 2019), the Theia OSO Land Cover Map 2017 (available at www.theia‐land.fr), and from BDTopo (IGN 2019). This was coupled with a more detailed regional inventory of hedgerows (from 2005 to 2008) and ponds (2012) and a national farming database from the EU LPIS (Land Parcel Identification System 2016) used to classify agricultural areas (see Table 1).

**TABLE 1:** Environmental variables used for species distribution modeling of each amphibian species in Pays de la Loire region. Associated references are available in the Appendix S2: Table S1

Variable category	Code	Variable description	Original resolution
Climatic	CLIM_1	First axis from a PCA on 12 worldclim variables and altitude	2.5 arc‐min/5 km
	CLIM_2	Second axis from a PCA on 12 worldclim variables and altitude	2.5 arc‐min/5 km
Land cover	%WOOD_DM	Proportion of deciduous and mixed forest	5 m
	%WOOD_C	Proportion of coniferous forest	5 m
	%CROP	Proportion of crop	20 m
	%PASTURE	Proportion of permanent pasture	20 m
	NB_PONDS	Pond density (or water point density)	5 m
	L_HEDGE	Hedgerow density	5 m
	L_ROAD_1ST	Primary road density outside urban areas	5 m
	L_ROAD_2ND	Secondary road density outside urban areas	5 m
	L_RIVER	Canal and river density	5 m
	%URBAN	Proportion of urban area	20 m

We calculated land‐cover variables in windows composed of a 500 m grid cell with a buffer of 300 m (see Table 1). This took into account landscape context based on species’ dispersal capacities as well as the resolution of the species dataset (Guisan & Thuiller, 2005). Distance and home range differ among amphibian species but a 1 km circle may be accepted as an average maximum range (Collins & Fahrig, 2017). Collins and Fahrig (2017) and Boissinot et al. (2019) show that landscape variables affect Anuran occupancy and diversity at this scale in agriculture‐dominated regions. We use the same environmental variables for all species (see Table 1) except *B. spinosus* for which “pond density” (water point <5,000 m^2^) was substituted by “water point density” because of this species’ ability to reproduce in larger water bodies with fish (Boissinot et al., 2019).

All predictive variables were centered and scaled. The spatial correlation between environmental predictors was investigated using the variance inflation factor (VIF) as a measure of multicollinearity and Pearson's correlation tests with VIF < 6 and r < 0.6 as advised by O’Brien, 2007 (see Appendix S2: Tables S2 and S3).

### 2.4 Habitat suitability modeling

#### 2.4.1 Statistical models

Different modeling algorithms can lead to varying results according to heterogeneous sensitivities and calculation processes (Thuiller et al., 2009). Therefore, consensus models based on multi‐modeling approaches (ensemble modeling) can improve final results by reducing “noise” associated with individual model errors (Araujo et al., 2005; Meller et al., 2014; Thuiller et al., 2009). For each species, we used one regression‐based approach (Generalized Additive Models, GAM) and one machine‐learning algorithm (Random Forest, RF) to predict and assess habitat suitability within the studied region with 50 bootstrap replicates. Presence points were randomly split 50 times into a training set (70% of the whole dataset) and the remaining 30% were used as testing set for internal evaluation (see 1.4). To construct these models, we used biomod2 package (Thuiller et al., 2009) in R environment v. 3.5.3 (R Development Core Team, 2019).

#### 2.4.2 Background data and pseudo‐absence selection

Modeling habitat suitability for a species with GAM or Random Forest requires both presence and absence data. In order to overcome the problem of missing absence data needed for most SDM, pseudo‐absence selection strategies have been developed to select absence data where real absence is most likely (Barbet‐Massin et al., 2012; Phillips et al., 2009). We tested three strategies for generating artificial absence points: (s1) simple random selection of background points within the studied region (Guisan et al., 2017); (s2) random pseudo‐absence selection excluding known presence points (Engler et al., 2004); (s3) random pseudo‐absence selection constrained to take sampling effort into account (see Appendix S3 for method). The latter strategy aimed to select pseudo‐absences where true absences were more likely. For this strategy, we considered three main sources of bias in pseudo‐absence selection: accessibility, linked to distance from roads or urban areas (Barbet‐Massin et al., 2012; Kadmon et al., 2004), attractiveness, relating to oversampling in protected sites or tourist areas (Phillips et al., 2009; Robinson et al., 2018) and observer effort, because certain administrative areas are covered by particularly active nature protection organizations (see Appendix S3). For each strategy, the number of artificial absences was fixed equal to the number of presence data (Barbet‐Massin et al., 2012; Liu et al., 2019) and we performed 10 replicates of the artificial absence generation processes.

#### 2.4.3 Ensemble modeling

Finally, we conducted ensemble modeling by calculating the median value of (1) all individual maps generated by GAM and Random forest (i.e. 500 maps/algorithm) (Thuiller et al., 2009) to compare internal versus external evaluation for each species. Second, we also calculated median values from ensemble maps calibrated with 100% of presence‐only data to compare different external evaluations sets (i.e. 10 maps/algorithm).

### 2.5 Internal and external model validation

We first use a cross‐validation method using a 30% random split of the whole set to asses each model (for pseudo‐absence selection strategies s1, s2 and s3) with 50 bootstraps repeated 10 times. We calculated the area under the curve (AUC) of a receiver operating characteristic (ROC) plot of the predicted model habitat suitability scores with (1) the 30% test dataset for internal validation and (2) with the larger filtered external independent dataset (e.g., *CS*.*2 + ABS + SUP*, see Figure 1) using Biomod2 package (Thuiller et al., 2009). AUC is the most common metric used in SDM studies, as it has the advantage of being threshold and prevalence independent and has been accepted as the standard measure for assessing SDM accuracy (Guisan et al., 2017). AUC > 0.50 signifies that the model has better prediction than a random model.

Second, we calculated AUC values, specificity (true negative rate), and sensitivity (true positive rate) of ensemble models calibrated with 100% of the presence‐only data using different evaluation sets derived from the global external dataset used in the previous stage (see Figure 1). These calculations (with 100 bootstraps) were performed using PresenceAbsence package (Freeman 2012) with a standard threshold value for presence–absence discrimination fixed at 0.5.

## 3 RESULTS

### 3.1 Model performance and selection

**FIGURE 2:**
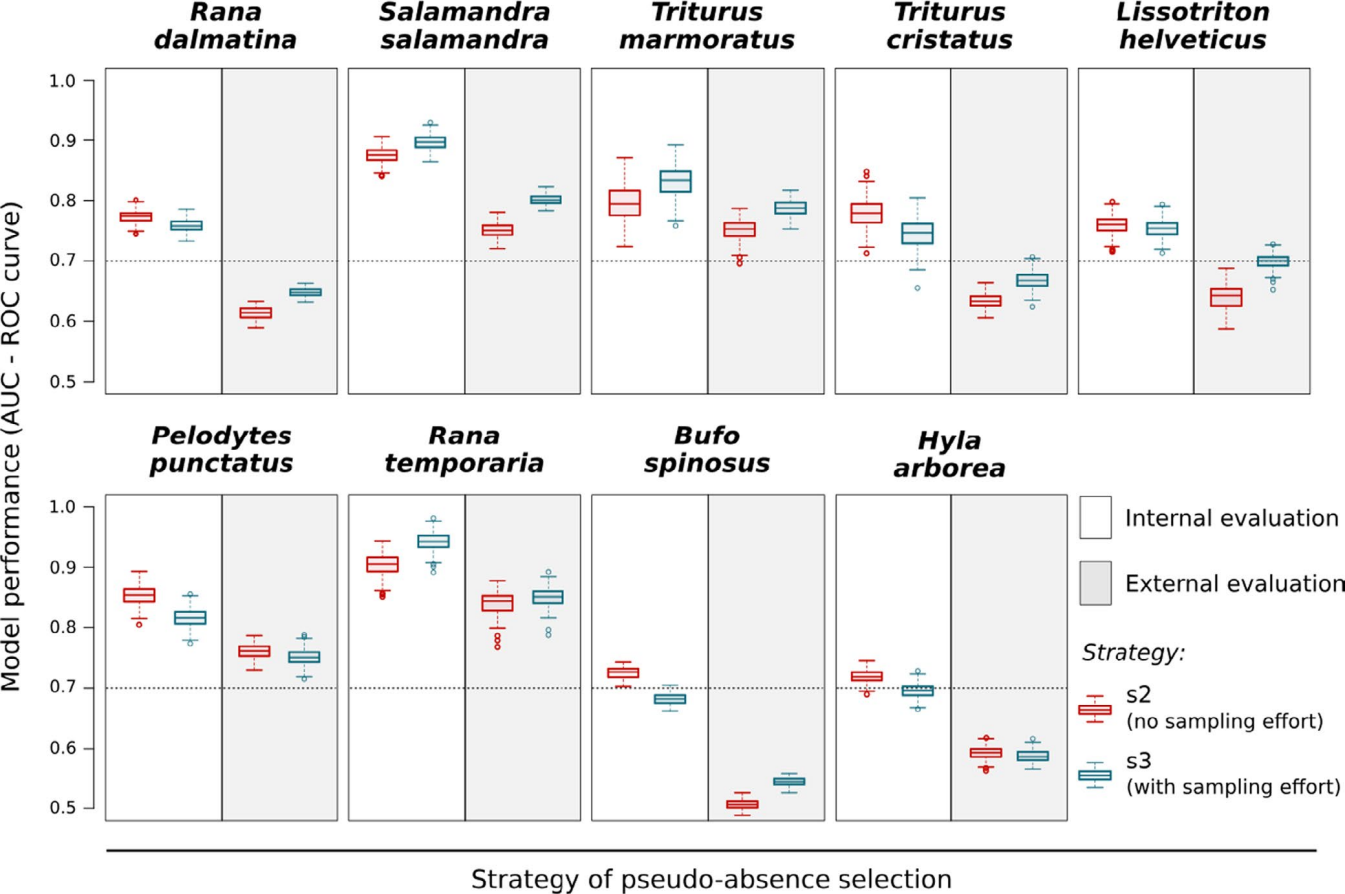
Model performance for the 9 studied species assessed by external or internal data using different pseudo‐absence selection strategies. Assessment by AUC under the ROC for GAM only are shown (see Appendix S1–S4). Artificial absence sampling strategies shown are s2 (random pseudo‐absence selection excluding known presence points) and s3 (random pseudo‐absence selection excluding known presence points and adjusted to consider site accessibility and sampling effort). Per strategy, 10 replicates of the artificial absence points generation processes with 50 bootstraps for the random selection of the straining set (70%) and the internal testing set (30%). Black dotted line indicates the 0.70 threshold above which models have an acceptable level of accuracy (Swets 1988)

For each species, the median AUC was higher with internal validation than external validation for all three pseudo‐absence selection strategies (s1, s2 and s3), both for GAM and Random Forest (see Figure 2 and Appendix S4) with a delta‐AUC ranging from 0.05 (*T. marmoratus*) to 0.21 (*B. spinosus*). With internal evaluation (cross‐validation), all models had excellent (AUC > 0.90) very good (0.80–0.90) or good accuracy (0.70–0.80) except for the model of *B. spinosus* and *H. arborea* including sampling effort parameters (s3). However, with external evaluation, only four species had a high level of accuracy (AUC > 0.70): *S. salamandra*, *T*. *marmoratus*, *P*. *punctatus,* and *R*. *temporaria*. For *R. dalmatina*, *T. cristatus,* and *L*. *helveticus*, model accuracies were poor (0.60 < AUC < 0.70) and for *B. spinosus* and *H. arborea* even poorer (AUC < 0.60). The strategy s1 (background data) was not selected neither with internal nor external evaluation.

The method used for pseudo‐absence selection influenced the predictive performance of models, but differences between AUC values were minimal (Figure 2). However, s2 (uncorrected sampling bias) was the best strategy for six species when internal validation was used, while s3 was best for seven species when external validation was used. Results for RF can be found in the supplementary material but do not differ greatly (Appendix S4: Figure S1).

### 3.2 Impact of model selection on final habitat suitability map

**FIGURE 3:**
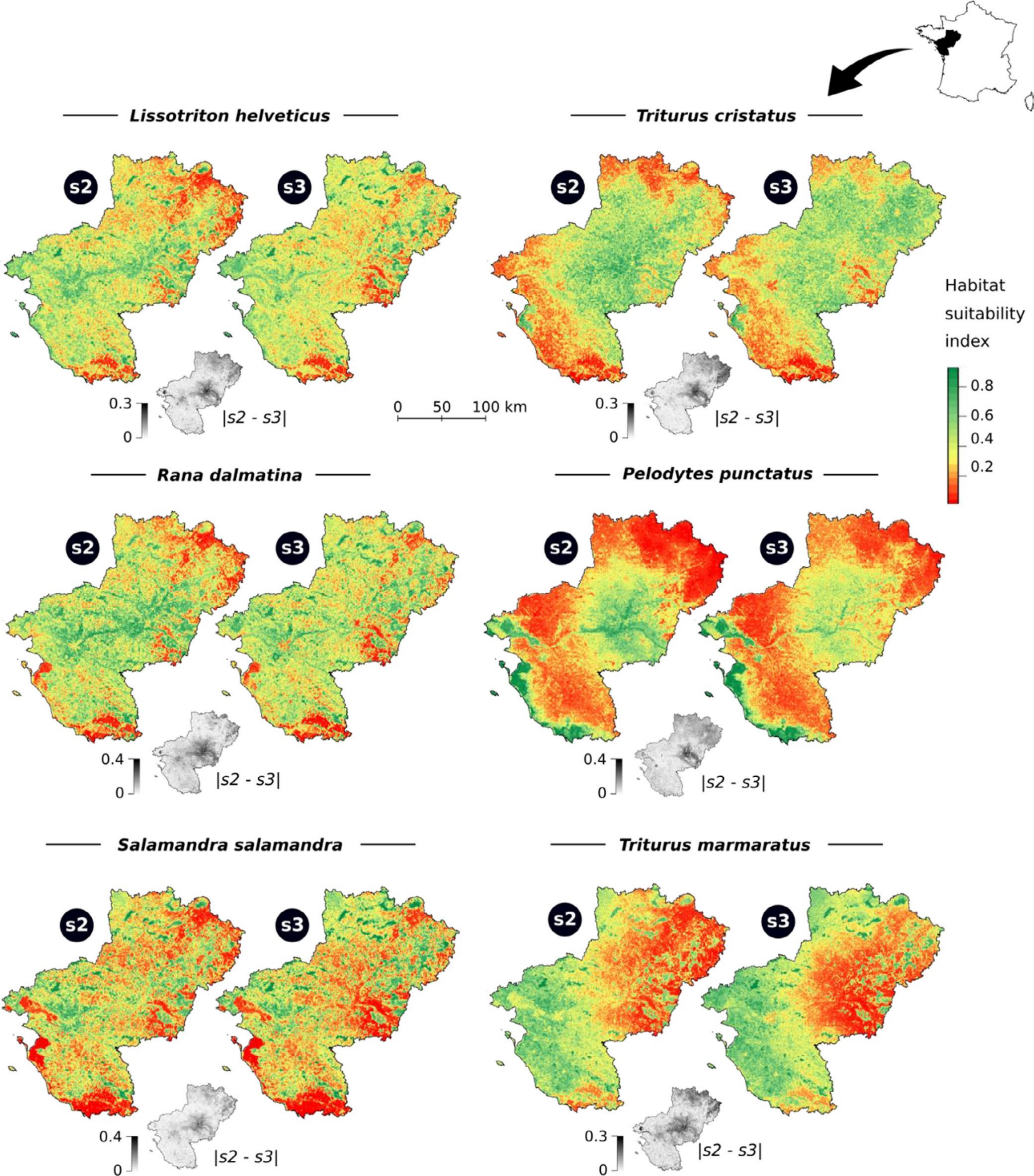
Habitat suitability maps for six studied species produced using two form of pseudo‐absence selection: s2 (random pseudo‐absence selection excluding known presence points) and s3 (random pseudo‐absence selection excluding known presence points and constrained to account sampling effort). The black and white map under each pair shows net difference between s2 and s3. Map resolution is 500 m

Internal or external validation resulted in different models being selected, based on AUC comparison. Therefore, the final habitat suitability maps selected by each of these two assessment methods would lead to different interpretation and conservation decisions (Figure 3). Maps for *H. arborea* and *B. spinosus* are not shown because of poor accuracy (see supplementary results Appendix S4). All response curves and associated variable contributions can be found in the supplementary material (Appendix S4: Figures S2 and S3).

### 3.3 Comparison of external evaluation sets

Values of AUC, sensitivity, and specificity to four species are shown in Table 2 (two Anurans and two Urodeles; one forest species and one generalist specie each). Results for other species and CS.1 (similar to CS.2) are presented in Appendix S4: Table S3. Considering AUC values, evaluation with the external dataset from participative science without filter data (*CS.0*) show more similar model selection results than internal cross‐validation except for *R. temporaria*. Sorting presence data led to decreased sensitivity and increased specificity for all species except for *S. Salamandra*. The models selected (s2 or s3) were similar for most species whether using stratified data from volunteers’ only (STRAT_CS) or stratified data with added professional observations (STRAT_ALL), or professional data only (PRO). See Table 2 and Appendix S4. We excluded the s1 model from the comparison because this model is never selected, either with internal or external evaluation.

**TABLE 2:** Model performance according to different filters and complementary fieldwork applied to the external evaluation dataset

		*Triturus marmoratus*			*Lissotriton helveticus*			*Hyla arborea*			*Rana temporaria*		
		SEN	SPE	AUC	SEN	SPE	AUC	SEN	SPE	AUC	SEN	SPE	AUC
*CS.0*	s2	0.63	0.41	0.58	**0.69**	0.29	0.53	**0.82**	0.43	0.68	0.71	**0.87**	0.87
	s3	**0.65**	**0.45**	**0.61**	0.64	**0.34**	0.54	0.78	**0.49**	0.68	**0.86**	0.84	0.88
*CS.2 + ABS*	s2	0.59	0.78	0.81	**0.67**	0.76	0.80	**0.71**	0.61	0.73	0.64	**0.88**	0.86
	s3	0.58	**0.87**	0.81	0.62	**0.88**	**0.86**	0.68	**0.64**	0.74	**0.82**	0.84	0.86
*CS.2 + ABS + SUP*	s2	0.58	**0.82**	0.82	**0.51**	0.77	0.68	**0.63**	**0.56**	0.63	0.59	**0.91**	0.85
	s3	**0.62**	0.78	0.81	0.49	0.78	**0.74**	0.57	0.54	0.63	**0.76**	0.86	0.85
*PRO*	s2	0.58	**0.83**	**0.85**	**0.41**	0.76	0.61	**0.59**	**0.39**	0.51	*0.50*	*0.95*	*0.71*
	s3	**0.79**	0.77	0.83	0.37	**0.79**	**0.64**	0.51	0.33	**0.54**	*0.50*	*0.92*	*0.71*
STRAT_CS	s2	0.71	**0.72**	**0.81**	0.67	0.81	0.82	0.72	0.64	0.73	0.79	0.57	0.69
	s3	0.70	0.69	0.78	0.68	0.82	**0.88**	0.71	0.63	**0.75**	**0.95**	**0.60**	**0.79**
STRAT_ALL	s2	**0.80**	**0.74**	**0.87**	0.66	0.76	0.78	**0.68**	**0.62**	0.70	0.84	0.57	0.73
	s3	0.78	0.70	0.83	**0.71**	**0.83**	**0.89**	0.66	0.60	**0.72**	**0.96**	0.58	**0.81**

Note

External datasets used were (see Figure 1): *CS.0* (all data from the standardized citizen science dataset); *CS.2 + ABS* (*CS.2* with 10% supplementary absence cells in very unfavorable habitats); *PRO* (data collected by professionals only in 2018–2019); *CS.2 + ABS + SUP* (citizen science data cited before adding all complementary fieldwork by professionals and volunteers); STRAT_CS (stratified data selection from *CS.2 + ABS* with complementary fieldwork by volunteers); STRAT_ALL (stratified data selection from *CS.2 + ABS + SUP*). Models assessed: s2 (random pseudo‐absence selection excluding known presence points) and s3 (random pseudo‐absence selection constrained to account sampling effort and correct sampling bias). SEN, sensitivity; SPE, specificity. Bold values show best values between s2 et s3 with delta >0.02 and italic values show species with less than 2 presence data. All analyses with a random sampling in presence selection with a distance condition or a stratified random selection were performed using 100 bootstraps (mean calculation).

## 4 DISCUSSION

External evaluation with independent data generated lower AUC values than cross‐validation, which calls into question the validity of models validated by commonly used selection threshold values such as AUC > 0.70. According to Araujo et al. (2005), internal evaluation with nonindependent data always leads to overoptimistic assessment of model performance. Even if cross‐validation is better than substitution procedures (Araujo et al., 2005; Edwards et al., 2006; Vaughan & Ormerod, 2005), split data for internal validation are nonindependent and do not avoid the main limits of correlative models in SDMs because of spatial or temporal autocorrelation, especially when sampling effort is highly heterogeneous (Edwards et al., 2006; Roberts et al., 2017). Our result supports criticisms of certain types of SDM and further highlights the need to be careful in their general interpretation and assessment (Lobo et al., 2008).

The difference between internal‐AUC and external‐AUC is particularly pronounced for the most common and generalist species in spite of the large number of available data, especially for *B. spinosus* and *H. arborea* whose models failed to attain an acceptable level of accuracy with external evaluation. Brotons et al. (2004) highlighted the difficulty of predicting distributions of the most generalist species. However, for such species, the use of filters increases specificity values considerably and the results are coherent with these species’ wide ranging and ubiquitous distributions. Using external presence–absence data also makes it possible to exploit the whole presence‐only dataset for calibration and to use stricter filters to reduce sampling bias or data culling to retain higher quality data (Isaak et al., 2014; Steen et al., 2019). Our study shows that it is possible to apply strong filters (e.g., STRAT_CS) but finally retain reasonable sample sizes for most species.

It should be noted that, for four species, *R. temporaria*, *T*. *marmoratus*, *S*. *salamandra,* and *P. punctatus*, our results were ambiguous. For the first three, all forest‐dwelling species or very closely related to woodlands (Boissinot et al., 2019), both internal and external validation methods selected models with sampling effort integrated (s3). Two main reasons could explain these results: first, presence data may have been insufficient for *R. temporaria*, and second these species’ affinity for forest habitats. *R. temporaria* is a rare species and is more dependent on wet forest, flood meadows, and small streams as breeding sites than other species (Boissinot et al., 2015). This species has a patchy distribution (i.e. locally abundant but regionally rare) and is difficult to detect. Hence, presence data are few in the studied area both in the opportunist dataset (*N* = 477 presence‐cells) and in the independent dataset (*N* = 13 presence‐cells). As highlighted by c, such factors can lead to model over fit, even with a relatively small number of variables, resulting in high AUC values. In addition, according to Brotons et al. (2004), low‐density habitat (i.e. forest habitat in our region) may be overweighted and it can be difficult to assess between good or bad suitability without adapted presence–absence data. Monitored forest sites are rare in our validation dataset, and the assumptions we used to define sampling effort may not be well adapted for forest specialists. Finally, *P. punctatus* is a rare species but abundant on the Atlantic coast. Unlike the other species, it is a pioneer, adapted to open areas, especially primary unvegetated habitats such as sand dunes and mudflats with frequent physical disturbance (Joly et al., 2005). These habitats are mainly located near the coast and along the main regional floodplains (Loire Valley), with a high local density of presence data. So this species is also patchily distributed and models may be affected by the same bias as *R. temporaria*. Alternatively, the similarity between AUC values may also relate to sampling effort bias along the Atlantic coast (e.g., Fithian et al., 2015). There results highlight the need to adapt methods and filters used for each species.

### 4.1 Using heterogeneous data from citizen science in SDM

Our results show that it is possible to obtain useful external and independent datasets for model validation from filtered standardized citizen science data. Indeed, the use of filters has successfully reduce bias and noise in citizen science datasets for SDM in others studies (Isaac et al., 2019; Robinson et al., 2018; Steen et al., 2019). In addition, filtered evaluation dataset showed coherent results according to Phillips et al. (2009). Indeed, choosing pseudo‐absence data with the same bias as occurrence data improved model performance.

Since external independent data are necessary for more robust assessment of SDM (Araujo et al., 2005), but prohibitive to collect, filtering low quality but large datasets from monitoring to obtain more standard and independent data may be worthwhile. In addition, AUC appears to be more informative when presence–absence data are used to assess and compare models than when presence‐background data alone is used (Jiménez‐Valverde, 2012). However, using detection–nondetection citizen data without filters may also lead to erroneous results because of overlapping sources of bias in both datasets (e.g., *CS.0* selects the same model as cross‐validation). The large amount of available data allows us to strongly select data according to our research objective. Our results using *PRO* datasets are inconclusive for rare species perhaps because their detection was insufficiently frequent (e.g., only two observations of *R. temporaria* for 132 sampled sites). Finally, we found that general rules to guide data sorting were difficult to define. Our results were sensitive to the type of data used, and the species studied, reinforcing the need to define filters according to available data and species’ ecology (Steen et al., 2019).

Independence between training and testing sets is an essential criterion, but data should also be unbiased or corrected. Selection methods have been developed to try to divide the opportunistic dataset strategically to increase the independence of the testing set for cross‐validation (see Block‐cross‐validation in Robert et al., 2017). However, this method does not make it possible to escape from the general biases linked to sampling effort and/or can create extrapolation problems (see Roberts et al., 2017). Using a more standardized dataset from a participatory science program (e.g., CS0) for the evaluation makes it easier to understand the sources of bias (presence of metadata and nondetection data), to better control them and to obtain more robust information on the absence data. However, these data may also share biases with the opportunistic dataset used for calibration. In our case, the sampling of the monitored sites (*CS0*) was partly biased towards volunteers’ place of residence and areas with a higher density of observers. These biases were reduced through additional sampling involving volunteers. Our results show that certain filters, as well as targeted complementary fieldwork, make it possible to reduce the biases identified and produce conclusive results. In addition, the use of a stratified sampling of the testing set along the suitability gradient from the SDM results (e.g., our STRAT_CS and STRAT_ALL datasets) appears to be a particularly interesting method showing stable and consistent results according to Phillips et al. (2009).

However, our method may be not applicable in all cases. In our study, external data came from a program with general population monitoring objectives, using standard methods designed to be accessible to a wide audience (e.g., novice and professionals). This program concerns all amphibians and their habitats, whereas many citizen science programs are limited to a particular taxonomic group or habitat type (cities, gardens or farms…) and would therefore be difficult to extrapolate to wider contexts.

### 4.2 Involved stakeholders and citizens in conservation research

Our study was part of a wider project for amphibian conservation in the Pays‐de‐la‐Loire region of western France. Involving citizens in the SDM evaluation process may make conservation action easier to implement, through both better shared knowledge and stronger personal involvement. Forrester et al. (2017) and Lewandowski and Oberhauser (2017) highlighted an increase in conservation advocacy among participants of citizen science projects that might improve access to evidence for conservationists and decision makers (Sutherland & Wordley, 2017). Maps are a specially a good tool for improving communication between researchers and volunteers in the context of citizen science (Zapponi et al., 2017). Indeed, many nature protection organizations are already involved in distribution atlas projects and naturalists are aware of data collection methods and local species distributions. They seek out ways to prioritize field observations; making a useful contribution to developing SDMs to guide conservation action can be a source of motivation, making scientist‐volunteer interactions easier.

## CONFLICT OF INTEREST

The authors declare that they have no financial conflict of interest with the content of this article.

## AUTHOR CONTRIBUTIONS

**Florence Matutini:** Conceptualization (equal); Data curation (equal); Formal analysis (lead); Funding acquisition (supporting); Investigation (lead); Methodology (lead); Visualization (lead); Writing‐original draft (lead); Writing‐review & editing (lead). **Jacques Baudry:** Conceptualization (equal); Methodology (supporting); Supervision (equal); Writing‐original draft (supporting); Writing‐review & editing (supporting). **Guillaume Pain:** Conceptualization (equal); Funding acquisition (lead); Methodology (supporting); Supervision (equal); Writing‐original draft (supporting); Writing‐review & editing (supporting). **Morgane Sineau:** Data curation (equal); Funding acquisition (supporting); Writing‐review & editing (supporting). **Josephine Pithon:** Conceptualization (equal); Funding acquisition (supporting); Methodology (supporting); Supervision (equal); Writing‐original draft (supporting); Writing‐review & editing (supporting).

## Supporting information

Appendix S1

Appendix S2

Appendix S3

Appendix S4

## Data Availability

Data sample and access procedure are available online: https://doi.org/10.5281/zenodo.4043460. Script and codes are available online: https://doi.org/10.5281/zenodo.4043460.
