# Supplementary material for: How citizen science could improve species distribution models and their independent assessment
Source: Ecol Evol. 2021 Mar 10;11(7):3028–39. doi: 10.1002/ece3.7210 (PMC8019030; doi:10.1002/ece3.7210)
Supplement: Supplementary file 2 — Appendix S2 [file ECE3-11-3028-s001.docx]

**Appendix 2 – Environmental variables: additional information**

| **Variable category** | **Code** | **Variable description** | **References** |
| --- | --- | --- | --- |
| Climatic | CLIM_1 | first axis from a PCA on 12 worldclim variables and altitude (see Table 4) | Rothermel and Semlitsch, 2002; Pineda and Lobo, 2009; Girardello *et al.*, 2010; Hartel *et al.*, 2010 |
|  | CLIM_2 | second axis from a PCA on 12 worldclim variables and altitude (see Table 4) |  |
| Land cover | %WOOD_DM | Proportion of deciduous and mixed forest | Pope, Fahrig and Merriam, 2000; Pellet, Guisan and Perrin, 2004; Cushman, 2006; Zanini *et al.*, 2008; Hartel *et al.*, 2010; Boissinot *et al.*, 2015; Zhang *et al.*, 2016; Collins and Fahrig, 2017; Boissinot, Besnard and Lourdais, 2019 |
|  | %WOOD_C | Proportion of coniferous forest |  |
|  | %CROP | Proportion of crop | Knutson *et al.*, 1999; Lehtinen, Galatowitsch and Tester, 1999; Mann *et al.*, 2009; Hartel *et al.*, 2010; Boissinot, Besnard and Lourdais, 2019 |
|  | %PASTURE | Proportion of permanent pasture | Scribner *et al.*, 2001; Janin *et al.*, 2009; Hartel *et al.*, 2010 |
|  | NB_PONDS | Ponds density | Janin *et al.*, 2009; Ribeiro *et al.*, 2011; Arntzen *et al.*, 2017; Boissinot, Besnard and Lourdais, 2019 |
|  | L_HEDGE | Hedgerow density | Joly *et al.*, 2001; Pellet, Guisan and Perrin, 2004; Vos *et al.*, 2007; Angelone, Kienast and Holderegger, 2011; Boissinot, Besnard and Lourdais, 2019 |
|  | L_ROAD_1ST | Primary Road density out of urban area | Vos and Chardon, 1998; Carr and Fahrig, 2001; Hels and Buchwald, 2001; Cushman, 2006; Eigenbrod, Hecnar and Fahrig, 2008; Hartel *et al.*, 2010; Petrovan and Schmidt, 2016; Boissinot, Besnard and Lourdais, 2019 |
|  | L_ROAD_2ND | Secondary Road density out of urban area |  |
|  | L_RIVER | Permanent rivers and canals density | Ficetola *et al.*, 2008 |
|  | %URBAN | Proportion of urban (build up) | Rubbo and Kiesecker, 2005; Gagné and Fahrig, 2007; Hartel *et al.*, 2010; Cayuela *et al.*, 2015; Zhang *et al.*, 2016 |

**Table 1. Environmental variables used for species distribution modelling of each amphibian species in Pays de la Loire region and associated references.** See Figure 2/ table 4 for the list of climatic variables.


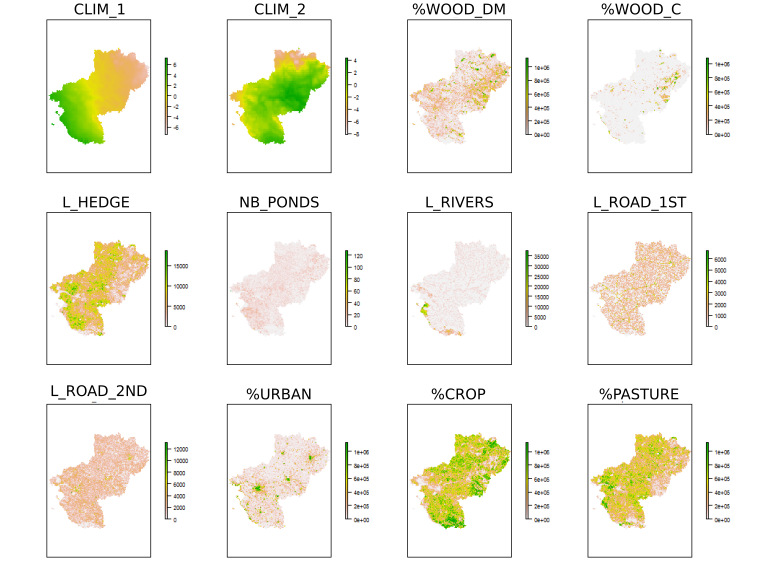


**Figure 1. Environmental variables used for modelling species distribution** (before scaling). Final maps resolution: 500m. CLIM_1 and CLIM_2 initial resolution was 5km².

| **Variables** | **VIF** |
| --- | --- |
| CLIM_1 | 1,26 |
| CLIM_2 | 1,09 |
| %WOOD_DM | 2,86 |
| %WOO8C | 1,89 |
| L_HEDGE | 1,89 |
| NB_PONDS | 1,18 |
| L_RIVERS | 1,27 |
| L_ROAD_1ST | 1,05 |
| L_ROAD_2ND | 1,18 |
| %URBAN | 2,66 |
| %CROP | 5,26 |
| %PASTURE | 4,67 |

**Table 2. Variance inflation factor values for each predictor**

**Table 3. Pearson correlation test for each predictor**

**Climatic variables**

**Table 4. variable selection and their contribution for each axis of the PCA**

**
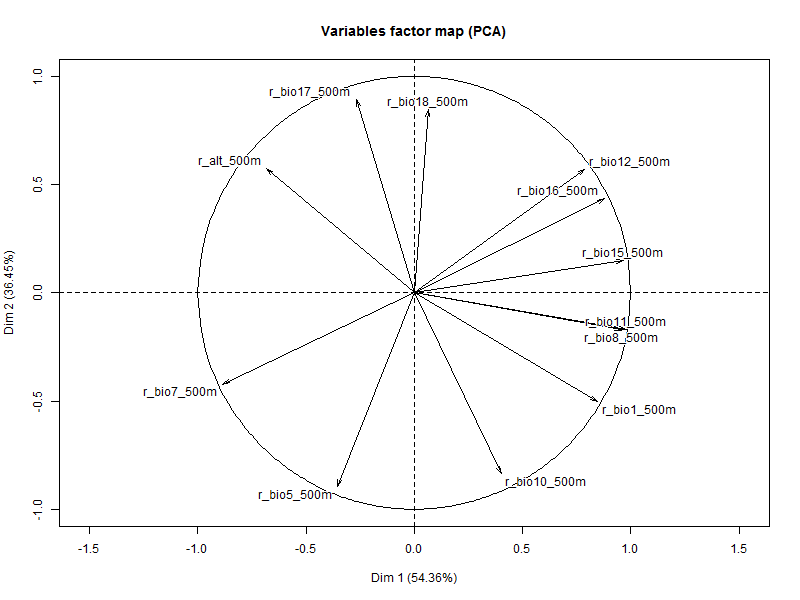
**

**Figure 2. Representation of the attributes on the factors 1 and 2 obtain by PCA by descriptor**. We use layer for Worldclim at regional extent to obtain 2 layers according to axis 1 (CLIM_1) and axis 2 (CLIM_2) of the ACP. Final resolution was 500m to homogenize pixels size of climatic variables and landscape variables layers.

**Bibliography**

Angelone, S., Kienast, F. and Holderegger, R. (2011) ‘Where movement happens: Scale-dependent landscape effects on genetic differentiation in the European tree frog’, *Ecography*, 34(5), pp. 714–722. doi: 10.1111/j.1600-0587.2010.06494.x.

Arntzen, J. W. *et al.* (2017) ‘Amphibian decline, pond loss and reduced population connectivity under agricultural intensification over a 38 year period’, *Biodiversity and Conservation*. Springer Netherlands, 26(6), pp. 1411–1430. doi: 10.1007/s10531-017-1307-y.

Boissinot, A. *et al.* (2015) ‘Small woods positively influence the occurrence and abundance of the common frog (Rana temporaria) in a traditional farming landscape’, *Amphibia Reptilia*, 36(4), pp. 417–424. doi: 10.1163/15685381-00003013.

Boissinot, A., Besnard, A. and Lourdais, O. (2019) ‘Agriculture , Ecosystems and Environment Amphibian diversity in farmlands : Combined in fl uences of breeding-site and landscape attributes in western France’, *Agriculture, Ecosystems and Environment*. Elsevier, 269(September 2018), pp. 51–61. doi: 10.1016/j.agee.2018.09.016.

Carr, L. W. and Fahrig, L. (2001) ‘Effect of Road Traffic on Two Amphibian Species of Differing Vagility’, *Conservation Biology*, 15(4), pp. 1071–1078. doi: 10.1046/j.1523-1739.2001.0150041071.x.

Cayuela, H. *et al.* (2015) ‘Highlighting the effects of land-use change on a threatened amphibian in a human-dominated landscape’, *Population Ecology*, 57(2), pp. 433–443. doi: 10.1007/s10144-015-0483-4.

Collins, S. J. and Fahrig, L. (2017) ‘Responses of anurans to composition and configuration of agricultural landscapes’, *Agriculture, Ecosystems and Environment*. Elsevier B.V., 239, pp. 399–409. doi: 10.1016/j.agee.2016.12.038.

Cushman, S. A. (2006) ‘Effects of habitat loss and fragmentation on amphibians: A review and prospectus’, *Biological Conservation*, 128(2), pp. 231–240. doi: 10.1016/j.biocon.2005.09.031.

Eigenbrod, F., Hecnar, S. J. and Fahrig, L. (2008) ‘The relative effects of road traffic and forest cover on anuran populations’, *Biological Conservation*, 141(1), pp. 35–46. doi: 10.1016/j.biocon.2007.08.025.

Ficetola, G. F. *et al.* (2008) ‘Influence of Landscape Elements in Riparian Buffers on the Conservation of Semiaquatic Amphibians’, 23(1), pp. 114–123. doi: 10.1111/j.1523-1739.2008.01081.x.

Gagné, S. A. and Fahrig, L. (2007) ‘Effect of landscape context on anuran communities in breeding ponds in the National Capital Region, Canada’, *Landscape Ecology*, 22(2), pp. 205–215. doi: 10.1007/s10980-006-9012-3.

Girardello, M. *et al.* (2010) ‘Models of climate associations and distributions of amphibians in Italy’, *Ecological Research*, 25(1), pp. 103–111. doi: 10.1007/s11284-009-0636-z.

Hartel, T. *et al.* (2010) ‘Amphibian distribution in a traditionally managed rural landscape of Eastern Europe: Probing the effect of landscape composition’, *Biological Conservation*, 143(5), pp. 1118–1124. doi: 10.1016/j.biocon.2010.02.006.

Hels, T. and Buchwald, E. (2001) ‘The effect of road kills on amphibian populations’, *Biological Conservation*, 99(3), pp. 331–340. doi: 10.1016/S0006-3207(00)00215-9.

Janin, A. *et al.* (2009) ‘Assessing landscape connectivity with calibrated cost-distance modelling: Predicting common toad distribution in a context of spreading agriculture’, *Journal of Applied Ecology*, 46(4), pp. 833–841. doi: 10.1111/j.1365-2664.2009.01665.x.

Joly, P. *et al.* (2001) ‘Habitat matrix effect on pond occupancy in newt’, *Conservation Biology*, 15(1), pp. 239–248. doi: 10.1046/j.1523-1739.2001.99200.x.

Knutson, M. G. *et al.* (1999) ‘Effects of landscape composition and wetland fragementation on frog and toad abundance and species richness in Iowa and Wisconsin, United States of America.’, *Conservation Biology*, 13(6), pp. 1437–1446. doi: 10.1046/j.1523-1739.1999.98445.x.

Lehtinen, R. M., Galatowitsch, S. M. and Tester, J. R. (1999) ‘Consequences of habitat loss and fragmentation for wetland amphibian assemblages’, *Wetlands*, 19(1), pp. 1–12. doi: 10.1007/BF03161728.

Mann, R. M. *et al.* (2009) ‘Amphibians and agricultural chemicals: Review of the risks in a complex environment’, *Environmental Pollution*. Elsevier Ltd, 157(11), pp. 2903–2927. doi: 10.1016/j.envpol.2009.05.015.

Pellet, J., Guisan, A. and Perrin, N. (2004) ‘A Concentric Analysis of the Impact of Urbanization on the Threatened European Tree Frog in an Agricultural Landscape’, *Conservation Biology*, 18(6), pp. 1599–1606. doi: 10.1111/j.1523-1739.2004.0421a.x.

Petrovan, S. O. and Schmidt, B. R. (2016) ‘Volunteer conservation action data reveals large-scale and long-term negative population trends of a widespread amphibian, the common toad (Bufo bufo)’, *PLoS ONE*, 11(10), pp. 1–12. doi: 10.1371/journal.pone.0161943.

Pineda, E. and Lobo, J. (2009) ‘Assessing the accuracy of species distribution models to predict amphibian species richness patterns’, *Journal of Animal Ecology*, 78(i), pp. 182–190. doi: 10.1111/j.1365-2656.2007.0.

Pope, S. E., Fahrig, L. and Merriam, H. G. (2000) ‘Landscape complementation and metapopulation effects on leopard frog populations’, *Ecology*, 81(9), pp. 2498–2508. doi: 10.1890/0012-9658(2000)081[2498:LCAMEO]2.0.CO;2.

Ribeiro, R. *et al.* (2011) ‘The pond network: Can structural connectivity reflect on (amphibian) biodiversity patterns?’, *Landscape Ecology*, 26(5), pp. 673–682. doi: 10.1007/s10980-011-9592-4.

Rothermel, B. B. and Semlitsch, R. D. (2002) ‘An Experimental Investigation of Landscape Resistance of Forest versus Old-Field Habitats to Emigrating Juvenile Amphibians’, *Conservation Biology*, 16(5), pp. 1324–1332. doi: 10.1046/j.1523-1739.2002.01085.x.

Rubbo, M. J. and Kiesecker, J. M. (2005) ‘Amphibian breeding distribution in an urbanized landscape’, *Conservation Biology*, 19(2), pp. 504–511. doi: 10.1111/j.1523-1739.2005.000101.x.

Scribner, K. T. *et al.* (2001) ‘Environmental correlates of toad abundance and population genetic diversity’, *Biological Conservation*, 98(2), pp. 201–210. doi: 10.1016/S0006-3207(00)00155-5.

Vos, C. C. *et al.* (2007) ‘Matrix permeability of agricultural landscapes: An analysis of movements of the common frog (Rana temporaria)’, *Herpetological Journal*, 17(3), pp. 174–182.

Vos, C. C. and Chardon, J. P. (1998) ‘Effects of habitat fragmentation and road density on the distribution pattern of the moor frog Rana arvalis’, *Journal of Applied Ecology*, 35(1), pp. 44–56. doi: 10.1046/j.1365-2664.1998.00284.x.

Zanini, F. *et al.* (2008) ‘Landscape effects on anuran pond occupancy in an agricultural countryside: barrier-based buffers predict distributions better than circular buffers’, *Canadian Journal of Zoology*, 86(7), pp. 692–699. doi: 10.1139/Z08-048.

Zhang, W. *et al.* (2016) ‘Responses of anuran communities to rapid urban growth in Shanghai, China’, *Urban Forestry and Urban Greening*. Elsevier GmbH., 20, pp. 365–374. doi: 10.1016/j.ufug.2016.10.005.
